# Effects of an Advance Care Planning Training Program for Certified Palliative Care Nurses in Japan

**DOI:** 10.1007/s13187-021-02125-9

**Published:** 2022-01-28

**Authors:** Yoko Yahiro, Mika Miyashita, Katsumi Nasu

**Affiliations:** 1 Department of Gerontological and Oncology Nursing, Graduate School of Biomedical and Health Sciences, Hiroshima University, Hiroshima, Japan; 2 Faculty of Nursing Department of Nursing, Fukuoka Jogakuin Nursing University, Fukuoka, Japan; 3 Department of Nursing, Faculty of Nursing Yasuda Women’s University, Hiroshima, Japan

**Keywords:** Advance care planning, Cancer patients, Certified palliative care nurse, Education program, Japan

## Abstract

Education programs for certified palliative care nurses who promote advance care planning (ACP) for cancer patients are important, but not currently available in Japan. This study aimed to develop an educational program of ACP for certified palliative care nurses in Japan and evaluate its effectiveness. A program consisting of four modules was implemented for 60 certified palliative care nurses in the Kyushu, Chugoku, and Shikoku regions. Participants attended one training session, and 39 responded to a survey on changes in ACP practice and difficulties with cancer nursing 3 months after the intervention. The Wilcoxon signed-rank test was performed to compare data before and after the intervention. The results obtained showed an increase in dialogue on ACP among patients/families and healthcare professionals (mean before the intervention = 24.49, mean after the intervention = 27.59, *p* = 0.045), and a significant decrease in the sense of difficulty with knowledge of and skills for ACP (mean before the intervention = 4.85, mean after the intervention = 4.30, *p* = 0.001). More than 90% of the participants gave positive comments on the evaluation items such as understanding and satisfaction with the educational program and appropriateness of the contents. After attending the training program, participants’ sense of difficulty with their knowledge of and skills for ACP decreased, and their practice of ACP increased. This program may promote the practice of ACP for cancer patients in the future.

## Introduction

Advance care planning (ACP) is a comprehensive process in which patients, their families, and healthcare professional share their concerns and values regarding future treatment and recuperation, and plan their care in preparation for a future decline in decision-making capacity [1]. ACP is characterized by the process of clarifying values and goals of care from the perspective of the patient’s life as a whole, and exploring how the patient will live in the future with the surrogate decision-maker and medical staff, rather than making individual decisions about treatment, life-prolonging procedures, and place of care [2]. ACP was introduced to Western countries in the 1990s, and not only increased patient satisfaction with care [3], but also reduced the mental burden on family members [4, 5]. It also reduced the cost of treatment for patients with advanced cancer [6].

Awareness of the importance of ACP is increasing in Japanese cancer medicine [7, 8]. Approximately 30% of doctors and nearly 20% of nurses recognize that they are actually practicing ACP [9]. A barrier to ACP is the lack of knowledge and skills, with only about 20% of physicians and nurses trained in decision support for terminally ill patients [9], and decision-making regarding end-stage medical care is centered on the family. In addition, some nurses are not skilled at communicating “bad news” to patients and their families [10]. In order to promote the practice of ACP in Japan, it is necessary to educate medical professionals about ACP so that these barriers can be resolved. The effects of educational programs on ACP have been reported worldwide. Participants in a training program utilizing a communication model increased their self-efficacy for ACP [11]. Furthermore, participants in a targeted training program received education using role playing and case studies, and, as a result, the implementation rate of ACP increased from that before training [12]. It is necessary for those who practice ACP to systematically learn the knowledge and skills related to ACP. Training programs on ACP in Japan are “The End-of-Life-Nursing Education Consortium-Japan” (ELNEC-J) and “Education For Implementing End-of-Life Discussion” (E-FIELD). However, there is no training program in Japan where nurses can systematically learn knowledge and skills related to ACP.

ACP for cancer patients is performed through a team approach, and certified nurses are involved in the practice, guidance, and consultation in a specific nursing field. Certified palliative care nurses are the cornerstone of multidisciplinary collaborations and facilitate the practice of ACP as the medical professional closest to a patient’s family. In order to apply ACP to more cancer patients in Japan in the future, it is essential to have certified palliative care nurses who have expertise in ACP palliative care, have high technical standards, and practice ACP as a team.

The purpose of this study was to develop a training program that enables systematic and practical learning of ACP in consideration of Japanese medical care and culture, and to determine the effectiveness of the training program developed for certified palliative care nurses who facilitate ACP.

## Methods

### Development of a Training Program

To select the contents of the training program, we reviewed ACP guidelines and models, training programs, and training programs related to communication [13–18]. The following topics were extracted: definition of ACP, knowledge of disease and treatment, ethics, legal basis, effectiveness of ACP, continuous follow-up, system construction, role of medical personnel, understanding of values, and communication. The program was structured to provide group education in 4 modules with 12 topics over 5 h in 1 day (Table 1). Modules 1 to 3 include basic knowledge of ACP and the role of nurses: NURSE (naming, understanding, respecting, supporting, exploring) [19]. A lecture was provided that included information on the communication model. NURSE is a model for communicating “bad news” at the time of cancer diagnosis, recurrence, or when aggressive treatment is difficult. It focuses on the emotions of the patient and family, with the goal of helping the patient cope with his or her own emotions. In Module 4, group work consisted of 5–6 people per group. In this training program, participants clarify and then resolve their issues in order to effectively practice ACP. Problem-based learning was used for decision-making in discussions with others. Module 4 was designed to allow participants to share their perceptions of barriers to ACP practice with group members, and to discuss solutions and goals for future action. All training was conducted by the researcher. All the training was conducted in person.

**Table 1:** Training programs

Module 1: Overview of ACP and the role of nurses (60 min Lecture)
•Definition and principle of ACP
•Basic concept of ACP
•Historical background of ACP
•Process of ACP
•Conditions of ACP
•Integrating ACP into practice
•Roles of nurses in ACP
•ACP and multidisciplinary care
**Module 2: Legal and ethical background of ACP (45 min Lecture)**
•Legal aspects of ACP
•Ethical aspects of ACP
•Patient’s right of self-determination and provision of information
•Legal basis for decision-making capabilities/abilities
•Advance directives and legal basis
•Adult legal guardian and medical consent
•Guardian
**Module 3: Communication process for ACP (45 min Lecture)**
•Communication in ACP
•The role of nurses to facilitate ACP
•Knowing the patient’s wishes
•Current settings of ACP
Conversations for initiating ACP
Appointing decision-makers
**Module 4: Examples of ACP and challenges (150 min Group discussion)**
•Current settings of ACP in Japan
•Current settings of ACP in other countries
ACP campaign
Using ACP workbooks on the website
•Challenges and measures for ACP
Reflection on one’s own practice
Challenges, background, and solution plans
Measures to achieve goals

### Participants

The selection criteria included certified palliative care nurses belonging to facilities in the Kyushu, Chugoku, and Shikoku regions, whose facility names and names were published on the official website of the Japanese Nursing Association. The sample size of the participants was planned to be a paired two-way ANOVA by setting an intervention group and a control group at the time of study planning. When calculated with effect size *f* = 0.25, *α* = 0.05, 1-*β* = 0.8, and scale number 2 with G * Power, a total of 34 or more samples were required in the intervention group and the control group. Although five study participants in the control group responded at T0, no one responded at T2. Hence, the pre-post test design was used for the intervention group, and the Wilcoxon signed-rank sum test was conducted to verify the effects of the intervention. As a result of setting effect size *f* = 0.5, *α* = 0.05, 1-*β* = 0.8 with G * Power, a sample size of 35 or more was required. The number of participants in this study was 39, which was sufficient for analysis.

### Procedure

Information was provided to 395 certified palliative care nurses belonging to facilities in the Kyushu, Chugoku, and Shikoku regions in a document describing the purpose of the study, the content and date of the training, the freedom to voluntarily participate in the study and withdraw consent, and the data encoding and protection of personal information after responses. Certified nurses who were willing to participate in training and cooperate in research entered their name, affiliation, and desired training date in the format created on the Web.

### Implementation of Training

Participants were presented with two candidate dates and selected the most convenient one.

### Data Collection

Data were collected at three time points: before training (baseline: T0), immediately after training (T1), and 3 months after training (T2). Before the start of the training day (T0), participants answered the questionnaire. Immediately after training (T1), the program was evaluated using the questionnaire survey. Three months after the training, participants completed a questionnaire that included the same items as before the training.

### Measures

#### Primary Outcome of Intervention: ACP Practice

Thirty-seven items to evaluate ACP for cancer patients were scored on a 5-point Likert scale (1: not practiced at all, 2: not practiced very much, 3: occasionally practiced, 4: sometimes practiced, 5: always practiced). Thirty-seven items are divided into 6 subscales: assessment of patients (4 items), dialogue on ACP among patients/families and medical staff (9 items), clarification of the values of patients (5 items), “facilitate proxy decision-makers’ participation in ACP” (4 items), decision-making support for treatment, including life-prolonging treatment (7 items), and ACP performance at the time of a reduced decision-making capacity (8 items). The scores for each item were summed, with higher scores indicating more frequent practice.

#### Secondary Outcome of Intervention: Difficulties with Cancer Nursing Practice

This scale was created by Onodera et al. [20] to assess the difficulties encountered by nurses practicing cancer nursing using a 6-point Likert scale (1: strongly disagree, 2: slightly disagree, 3: disagree, 4: agree, 5: slightly agree, 6: strongly agree), with higher scores indicating greater difficulty. It consisted of 5 items and 20 sub-items related to ACP, out of 6 items such as communication, knowledge/skills, doctor’s treatment/response, medical condition explanation/notification, and system/cooperation. The *α* coefficient of each item was 0.69 to 0.74.

#### Secondary Outcome of Intervention: Stages of Change Model

The stages of change model is a model that categorizes the process of people changing to a desired behavior into five stages. Based on this model [21], we asked the subjects to select the most appropriate response: “I am not thinking of starting within 6 months,” “I am thinking of starting within 6 months,” “I am thinking of starting within 1 month,” “I have been doing this for less than 6 months,” and “I have been doing this for more than 6 months.

#### Evaluation of the Training Program

Six items were created to evaluate the program structure and training method. In each module, (1) understanding of the content, (2) satisfaction with the content, (3) clarity of the goal, (4) the appropriateness of the content to achieve the goal, (5) appropriateness of the method, and (6) utility for the future practice of ACP were evaluated on a 5-point Likert scale (1: strongly disagree, 2: slightly disagree, 3: agree, 4: slightly agree, 5: strongly agree).

### Statistical Analysis

After calculating descriptive statistics for each item, the Wilcoxon signed-rank test was performed to compare the ACP practice scale, difficulty scale for nurses regarding cancer nursing, and T0 and T2 data from the stages of change model. The significance of differences was set at *P* < 0.05.

## Results

### Participant Characteristics

Daily training was performed twice with 18 and 42 certified nurses in each session. Forty-four out of the 60 participants who underwent the training program responded to the survey 3 months later. We analyzed the responses of 39 participants (65%), excluding those with missing values. The average age of participants was 43.21 ± 6.45 years, and the average years of experience of certified palliative care nurses was 5.05 ± 3.15 years (Table 2).

**Table 2:** Participant characteristics

	Participants	
	***X***	**SD**
Age (years)	43.2	6.45
Years’ experience as a certified palliative care nurse	5.0	3.15
	***n***	**%**
**Sex**		
Female	37	95
Male	2	5
**Work environment**		
Palliative care team, full-time	13	33.0
General ward	12	31.0
Palliative care unit	7	18.0
Outpatient department	3	8.0
Admission and discharge assistance	1	2.5
Visiting nurse services	1	2.5
Others	2	5.0
**Official position**		
Staff	21	54.0
Director	16	41.0
Others	2	5.0
**Workshop experience**		
Yes	13	33.3
**Educational background**		
Nursing school	31	79.0
Junior college	3	8.0
University	5	13.0

### ACP Practice

In ACP, the average value of T2 in “Dialogue on ACP among patients, family members, and medical staff” was significantly higher than the average value of T0 (*p* = 0.045). “Assessment of patients,” “Decision-making support for treatment, including life-prolonging treatment.” No significant differences were observed between the mean T0 and mean T2 of “confirmation of hope,” “promotion of the participation of surrogate decision-makers,” and “support for patient decision-making regarding life-prolonging treatment” (Table 3). Coefficient alpha was “Assessment of patients: 0.76,” “Dialogue on ACP among patients/families and medical staff: 0.72,” “Facilitate proxy decision-makers’ participation in ACP: 0.77,” “Decision-making support for treatment, including life-prolonging treatment: 0.78,” and “ACP performance at the time of a reduced decision-making capacity: 0.70.”

**Table 3:** Nurses’ difficulty scale for caring for cancer patients after ACP practice

	Baseline (T0)		After 3 months	*P-*value
	Mean (SD)		Mean (SD)	
**ACP practice or performed (37 items)**				
Factor 1: Assessment of patients (4)	13.95 (4.48)		14.79 (4.03)	0.324
Factor 2: Dialogue on ACP among patients/families and medical staff (9)	24.49 (11.19)		27.59 (9.90)	0.045
Factor 3: Clarification of patient values (5)	20.46 (3.51)		20.59 (2.84)	0.742
Factor 4: Facilitate proxy decision-makers’ participation in ACP (4)	11.36 (3.78)		12.33 (3.66)	0.078
Factor 5: Decision-making support for treatment, including life-prolonging treatment (7)	27.00 (5.40)		27.82 (5.43)	0.509
Factor 6: ACP performance at the time of a reduced decision-making capacity (8)	24.23 (10.75)		25.97 (9.68)	0.741
**Difficulties of nurses practicing nursing for cancer patients (20 items)**				
Communication (5)	22.00 (3.76)		21.76 (3.92)	0.464
Knowledge and skill (1)	4.85 (0.96)		4.3 (1.00)	0.001
Collaboration with doctors (4)	16.02 (3.85)		16.17 (3.80)	0.679
Disclosure and explanation (4)	15.41 (3.27)		14.66 (3.40)	0.134
Hospital system and regional alliances (6)	27.12 (4.20)		26.66 (5.02)	0.47

### Difficulties with Cancer Nursing Practice

The average value of T2 for “knowledge/skills” was significantly lower than the average value of T0 (*p* = 0.001). “Communication,” “doctor’s treatment/response,” “medical condition explanation/notification.” No significant differences were observed between the average values of T0 and T2 for “System/Cooperation” (Table 3).

### Stages of Change Model

The grades of the stages positively changed from T0 to T2 in 19 (48.7%) participants, remained unchanged in 14 (35.9%), and negatively changed in 6 (15.4%).

### Evaluation of the Training Program

Participants were asked about “understanding of the contents,” “satisfaction with the contents,” “clarity of the goals of the module,” “appropriateness of the contents to achieve the goals,” “appropriateness of the method,” and “future” for all modules. More than 90% of participants provided positive responses for “usefulness of ACP in practice.”

## Discussion

This is the first study in Japan to develop a training program for certified palliative care nurses who practice ACP for cancer patients and clarify its effects. The implementation of the training program increased the dialogue on ACP among patients/family members and medical personnel, which was consistent with previous findings [11, 12]. A large part of the ACP process consists of dialogue between the patient/family and healthcare professionals, which is an important component of ACP [12, 14, 15]. Increased dialogue about ACP between participants and patients/families may help promote ACP.

The use of communication models is effective for medical professionals to promote ACP with patients [12, 14, 15]. NURSE, which was adopted in this training program, is used to convey “bad news” and subsequently provide support [19]. In the study, as the participants listened to the patients and their families with reference to the NURSE, the patients’ families were able to express their own ideas.

Increased dialogue on ACP among participants and medical professionals contributes to the promotion of ACP in team medical care. ACP for cancer patients in Japan is promoted within the context of team medicine, and nurses play a central role in the team [8], which contributes to the resolution of ACP barriers caused by a lack of communication between teams and departments.

Regarding the difficulties of cancer nursing, the value for “I feel that my knowledge of and skills for ACP are insufficient” was lower 3 months after training than before the training and was attributed to the training program. Therefore, certified nurses recognized that they had acquired knowledge of and skills for ACP. Among the 39 certified nurses who participated in this training program, 26 (66.7%) systematically learned about ACP for the first time. Previous studies also reported that obtaining information on and skills for ACP improved the practice rate of ACP [11, 12].

In the “stages of change model,” which surveyed the awareness of certified palliative care nurses of behavioral changes toward ACP, 19 (48.7%) changed to a more positive stage from before training. Thus, knowledge of ACP gained in this training increased the awareness of changing behavior and motivated the participants to take action.

Motivation is important for behavioral changes [21]. Participants conducted group work in training, shared barriers related to ACP, and discussed their solutions and future action goals. More than 90% of the respondents answered positively about the training method and its effectiveness for future utilization; therefore, group work discussions appeared to raise participants’ awareness of ACP and motivated behavioral changes. In the future, it will be necessary to incorporate role playing and simulations of different situations and to consider practical training contents and methods that will promote the practice of ACP.

### Research Limitations

The present study has some limitations. Participants in the training program were certified palliative care nurses who belong to facilities throughout Japan, and the sample size is small. In addition, this study had no control group. Therefore, the results obtained cannot be generalized. Further studies are needed to clarify the effectiveness of the program by expanding the target area and implementing the program for a larger number of subjects with using a randomized controlled trial design. Moreover, the present study only investigated the effects of training for up to 3 months after the program. Further studies to evaluate the long-term effects of this training program are needed. In addition, since the number of subjects in the present study was small, it was not possible to perform an analysis that divided data into subgroups or that considered related factors. Another limitation is that the evaluation indicators used in this study did not accurately reflect the effects of the program; therefore, an appropriate evaluation index needs to be developed.

Since the program was for certified palliative care nurses, there may have been little change after taking the program. However, when educating nurses who are not engaged in palliative care, it may be necessary to change the contents and methods of the program. In the future, we would like to develop this program so that nurses who are not engaged in palliative care will also receive ACP training.

## Data Availability

The datasets generated and/or analyzed during the present study are available from the corresponding author upon reasonable request.

## Code Availability

Not applicable.

All procedures performed in studies involving human participants were in accordance the ethical standards of institutions and/or national research committees, and the 1964 Helsinki Declaration and subsequent amendments or equivalent ethics. It complied with the standards.
